# Advances in Silicon-Based UV Light Detection

**DOI:** 10.3390/mi16101130

**Published:** 2025-09-30

**Authors:** Arif Kamal, Seongin Hong, Heongkyu Ju

**Affiliations:** 1 Department of Semiconductor Engineering, Gachon University, Seongnam-si 13120, Republic of Korea; arifkamal37@gachon.ac.kr; 2 Gachon Bionano Research Institute, Gachon University, Seongnam-si 13120, Republic of Korea; 3 Department of Physics and Semiconductor Science, Gachon University, Seongnam-si 13120, Republic of Korea

**Keywords:** UV photodetector, silicon, wide bandgap, responsivity, surface engineering

## Abstract

Silicon (Si), the cornerstone semiconductor in the micro-electronics industry, can provide a cost-efficient platform with mature technologies for photodetection in visible and near-infrared regions. However, its intrinsic properties, such as a narrow bandgap and the shallow penetration depth of ultraviolet (UV) light into its surface with surface trap states, remain challenges, rendering it unsuitable for effective UV light detection. Various techniques have been reported to circumvent these surface defect-induced difficulties. In addition, wide-bandgap semiconductors that favor UV light absorption in a solar-blind way have been combined with Si for UV light detection in order to retain the device’s compatibility with Si-CMOS processes, though it still faces challenges that need to be overcome. This review starts with concepts of basic parameters of photodetectors and categorizes UV photodetectors according to their detection mechanisms. We also present a review of wide-bandgap semiconductor-based UV light detectors and those based on Si, with a discussion of surface defect minimization. In addition, we review the hybrid structure of the two kinds, i.e., wide-bandgap semiconductors and Si, and discuss their properties that produce synergistic effects. Lastly, we provide conclusions and outlooks for the possible development of next-generation UV light detectors based on Si.

## 1. Introduction

**Figure 1:**
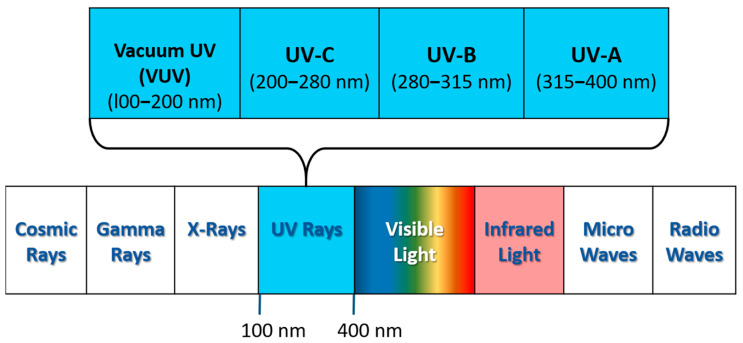
The spectral range of electromagnetic radiation.

Ultraviolet (UV) radiation occupies the invisible band of the electromagnetic spectrum, with wavelengths ranging from 100 to 400 nm. UV radiation is further classified into four categories, namely, UV-A (315–400 nm), UV-B (280–315 nm), UV-C (200–280 nm), and Vacuum UV (VUV) (l00–200 nm). The sun is one of the sources of UV light, though substantial amounts of its light are screened out via the absorption of atmospheric gases [1]. The full spectrum of electromagnetic waves is depicted in Figure 1.

UV light has found applications ranging from water purification [2,3,4,5] and photolithography [6,7,8,9,10,11] to optical communication [12,13], due to its high photon energies and lower degree of diffraction. Such a variety of applications of UV light requires its accurate and precise detection, generating continued interest in the development of highly efficient UV detectors. The numerous UV detection systems that have been developed can be categorized into photoelectric effect-based photomultiplier tubes [14], photoconductive detectors [15,16], photovoltaic detectors [17,18], field-effect transistors (FETs) [19,20,21], pyroelectric detectors [22,23], and thermoelectric detectors [24,25]. UV detectors usually employ wide-bandgap semiconductors that generate photogenerated electrons and holes only when the incident photon energy is equal to or greater than their bandgap energies. These wide-bandgap semiconductors include inorganic compound semiconductors [26,27,28,29,30], organic materials [26,27,28,29,30,31,32,33], and perovskite materials [34,35]. The wide bandgap energy required for UV photon absorption can also be realized by reducing the size of photon-absorbing material into nanoscales, such as quantum wells [36], quantum wires [37,38], and quantum dots [39,40,41].

Silicon (Si), widely used in semiconductors for detecting visible light, has a bandgap energy of about 1.12 eV at room temperature [42,43] and a relatively long carrier diffusion length of hundreds of micrometers [44,45]. However, its narrow bandgap energy allows it to absorb photons mostly in the visible and IR regions, leading to solar sensitivity. Moreover, the UV penetration depth into Si’s surface is known to be around 10 nm, resulting in photogenerated carriers near the surface that cannot diffuse into the bulk, but are annihilated through carrier recombination via surface defects and traps [46]. Furthermore, an unwanted oxidized layer of SiO_2_ on Si’s surface can absorb or scatter UV photons, with the consequence of rolling back UV light absorption by Si [47,48].

**Figure 2:**
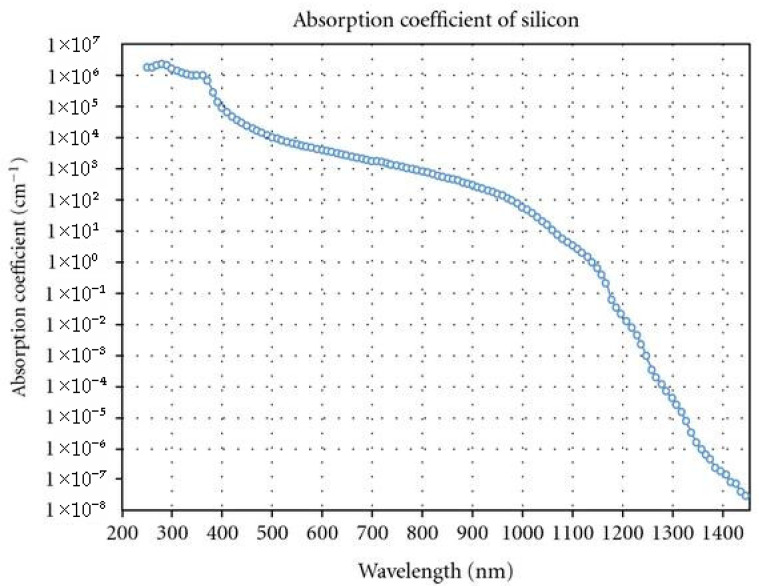
Wavelength-dependent absorption coefficient of silicon. Reproduced from [51] (*Journal of Nanotechnology*, 2012), licensed under CC BY 4.0.

Despite the abovementioned drawbacks of Si for UV light detection, it possesses a considerably large absorption coefficient of over 10^6^ cm^−1^ in the UV range. Figure 2 presents the absorption coefficient of Si as a function of wavelength, ranging from the 200 nm (UV) to 1500 (IR) region. Its strong absorption in the UV region makes it inherently fit for UV detection. In addition, its compatibility with the pre-matured technologies of complementary metal oxide semiconductors (CMOSs) favors mass production of Si-based UV detectors at a low cost due to their lower power consumption and their easy integration into a system (e.g., system on a chip) [49,50]. These aspects, together with other encouraging characteristics of Si, including the long lifetime of photogenerated carriers and its abundance in nature, have tempted researchers to develop Si-based UV detectors. This review article is structured as follows. Section 2 presents the definition of several parameters relevant to the performance of photodetectors that were not restricted to those for UV detection. Section 3 provides a classification of UV photodetectors according to detection mechanisms. Section 4 briefly reviews photodetectors based on wide-bandgap semiconductors, along with their performance. Section 5 covers Si-based photodetectors, along with their properties and progress made to overcome the relevant challenges. The hybrid structure of wide-bandgap semiconductors and Si is also presented with a relevant discussion. Lastly, we provide conclusions and outlooks for the future direction of Si-based UV light detectors.

## 2. Parameters Relevant to Photodetector Performance

A photodetector, in general, is characterized by a set of parameters that determine its performance in detecting incident light power. These parameters include the dark current (I_d_), total current (I_t_), photocurrent (I_p_), applied voltage bias, responsivity (R), external quantum efficiency (EQE), spectral response (SR), response time (t_r_), signal to noise ratio (SNR), noise equivalent power (NEP), specific detectivity (D), and linearity and dynamic range. In this section, we define the abovementioned parameters as follows:

i. **Dark Current (I_d_)**

Dark current (I_d_) is the current that flows through the photodetector circuit at a given voltage bias when no light is incident on the detector active area. It arises primarily due to thermally excited carriers through mid-gap trap states, including those near the surface or the interface of a light-absorbing semiconductor/medium. I_d_ restricts the photodetector sensitivity, thus being considered one of the most significant parameters for detecting light with low optical power [52,53].

ii. **Photocurrent (I_ph_)**

Photocurrent (I_ph_) is the current obtained by subtracting dark current from the current measured when light is incident on a photodetector area. I_ph_ should have no dependence on ambient temperature but be directly in proportion to the optical power of light incident on the active photodetector area. When subtracting dark current for estimating I_ph_, light absorption-induced thermal energy dissipation that may change the level of dark current must be considered.

iii. **Responsivity (*R*)**

Responsivity (*R*) is the ratio of the I_ph_ with respect to the power of incident light ($P_{in}$), thus having a unit of Ampere/Watt. It manifests how effectively a photodetector converts optical power to electrical current. Particularly, in photodetectors with no internal gain such as in photodiodes, *R* is directly proportional to internal quantum efficiency η, representing a probability that a photo-generated pair of charge carriers are collected per unit photon absorbed, as given by

(1) $$\eta =(I_{ph}/e)/(P_{a}/\hbar \omega )=(\hbar \omega /e)(R/A),$$

where e is the electronic charge, $P_{a}$ is the absorbed light power, ω is the frequency of absorbed light, ℏ is the Plank constant, and $P_{a}=A\;P_{in}$ (A<1 is the absorption efficiency). In general, η depends on the materials and structure of a photodetector.

Typically, R<1, and it becomes larger at a longer wavelength since there is a greater number of photons present in 1 W optical power at longer wavelengths. Meanwhile, R can exceed unity in some cases, including a photodiode with a very large η value (e.g., close to unity) at a longer wavelength, for instance, near infrared. In addition, internal gain available from multiple charge carriers generated from absorbed single photon can account for R>1, such as in photoconductive detectors and avalanche photodiodes.

iv. **External Quantum Efficiency (EQE)**

External quantum efficiency (EQE) describes how effectively incident photons are converted to photogenerated carriers in a photodetector, taking into account the optical losses of incident light due to light reflection/scattering from the surface of the detector’s active medium and light transmission through it. This allows EQE to be given by the following:

(2) EQE=ηA.

Similarly η, EQE can exceed unity (100%) in cases of internal gain due to the contribution of multiple carriers to current per single photon absorbed.

v. **Voltage Bias**

**Figure 3:**
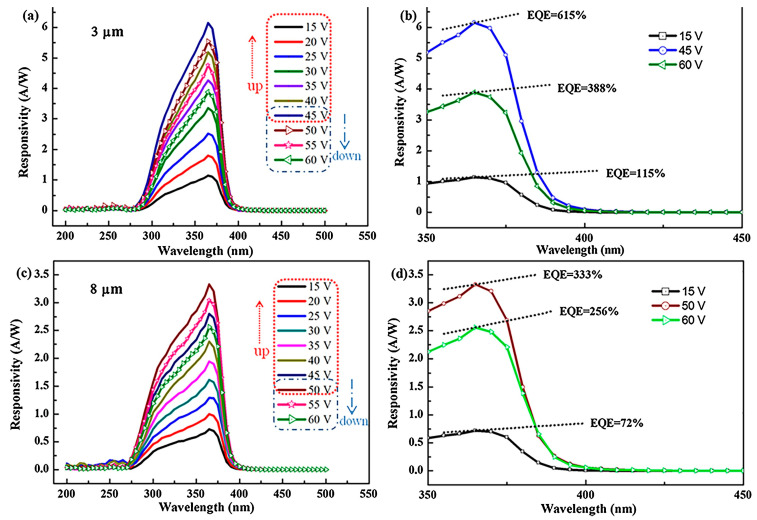
Responsivity spectra of ZnO thin films based UV photodetector with interdigitated electrodes for various bias voltage. (**a**,**b**) are for 3 μm electrode spacing, while (**c**,**d**) are for 8 μm electrode spacing. Reproduced with permission from [59] (Sensors and Actuators A: Physical, Elsevier 2018).

The voltage bias is the external voltage applied across the electrode of a photodetector to separate photogenerated electrons and holes in typical photoconductive detectors or even accelerate them into hot carriers with huge kinetic energy, such as in an avalanche photodiode. Figure 3a,b show the *R* versus UV wavelength for various bias voltages, while Figure 3c,d show the EQD versus UV wavelengths for various bias voltages in a photodetector. As the bias voltage rises from zero, *R* and EQE increase until a given bias voltage. This is simply due to the fact that enhanced fields increase the drift current but suppress the radiative recombination rate. However, when increasing the bias voltage beyond the given point, defect state-mediated non-radiative recombination begins to play a significant role due to a widened depletion region in photodiodes such as Shockley–Read–Hall recombination [54,55] and trap-assisted tunneling [56,57,58].

vi. **Response Time**

Photodetector response time refers to the time it takes for photocurrent to rise or fall when light is turned on or off, being inversely proportional to the device bandwidth. Response time of the order of magnitude of ns or ps can support high-speed optical signal processing. Many factors affect the response time, such as traverse time of photogenerated carriers across the device electrodes, carrier mobility, carrier diffusion time, carrier lifetime, the device resistance-capacitance product, and carrier trapping/non-radiative recombination [60,61,62].

vii. **Noise Equivalent** **Optical Power (NEP)**

Noise equivalent optical power (NEP) refers to the incident optical power that produces a signal-to-noise ratio of unity, and scales with the square root of the electrical noise power. The electrical noise is comprised mainly of shot noise, dark current noise, and Nyquist noise—thus, NEP scales with the square root of the device bandwidth Δf (Hz).

viii. Specific Detectivity (D*)

Detectivity (D) is defined by the reciprocal of NEP, representing the photodetector sensitivity. However, NEP scales with $\sqrt{\Delta f}$ and $\sqrt{A}$, where A is the photodetector active area to which dark current is approximately proportional. Therefore, NEP can be normalized by $\sqrt{(\Delta f)A}$ into a parameter independent of the active area size and bandwidth. Then, the specific detectivity (D*) is defined by the reciprocal of the normalized NEP so that D* $=\sqrt{A\Delta f}/NEP.$ It is the figure of merit expressed in the unit of Jones ($\equiv cm\sqrt{Hz}/W$), which is used for estimating how low optical power of light can be detected regardless of the size of the photodetector active area and its bandwidth. In cases where dark current noise dominates electrical noise, such as in the detection of low light power and infrared light detection, D* can be approximated as follows:

(3) $$D*\;≅R\sqrt{A/2eI_{d}}.$$

## 3. Classification of Photodetectors by Detection Mechanism

The development of UV photodetectors has evolved into a diverse family of devices, each customized for a particular spectral region and sensitivity. In this section, we categorize them according to light-detection mechanisms, though this classification may equally apply to photodetectors for visible and infrared light wavelengths. This classification may help us to estimate which types of photodetectors would be favorable for UV light detection in terms of suppression of dark current, optimization of responsivity, and internal gain harvest through carrier multiplication (see Table 1 and Table 2).

### 3.1. Photomultiplier Tube (PMT) for Photodetection

**Figure 4:**
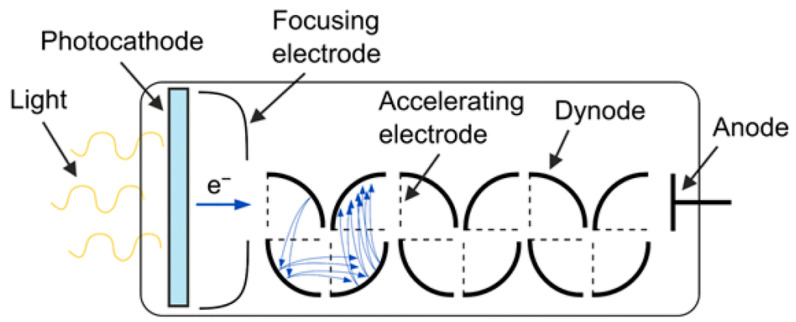
A schematic of a typical PMT, illustrating sequential processes, photoelectron ejection by a photoelectric effect, photoelectron acceleration for acceleration (dashed line), secondary electron emission at a dynode, the following cascading processes for multiple electrons generation/their acceleration, and their collection by an anode. Reproduced with permission from [71] (*Applied Physics B: Lasers and Optics* 2019).

Electromagnetic waves in a wide spectrum ranging from deep UV to near infrared can be detected using the photoelectric effect, whereby electrons are ejected from the material surface when UV photons are incident on it. Such photoelectrons are generated when a photon energy exceeds the work function/electron affinity of the material, which acts as a photocathode, as shown in Figure 4. A voltage as high as hundreds of volts is applied between a photocathode and a dynode, subsequently accelerating photoelectrons toward it. They eventually hit a dynode, generating secondary electrons via impact ionization, e.g., inelastic collision whereby bound electrons in a dynode are knocked out into the vacuum (secondary electrons). The secondary electrons are then accelerated and hit the next dynode, leading to cascading generation of hot electrons, which are finally collected by the anode of the detector. The cascading multiplication of photoelectrons in this kind of device, called a photomultiplier tube (PMT), accounts for electrical signal gain, which could grow exponentially to be enormous enough to detect the single-photon level. Despite this ultrahigh sensitivity, PMTs face challenges in their miniaturization and robustness. PMT finds various applications in fluorescence, nuclear/high-energy physics, and medical imaging [63,64,65]. For the detection of deep UV and daylight UV, alkali metal compounds such as CsI and Ce_2_Te can be used for a photocathode of PMT [66,67,68]. Moreover, bi-alkali metal compounds such as K_2_CsSb can be used for a photocathode of PMT that detects near-UV and blue light [69,70].

### 3.2. Photodiodes and Photovoltaic Devices for Light Detection

**Figure 5:**
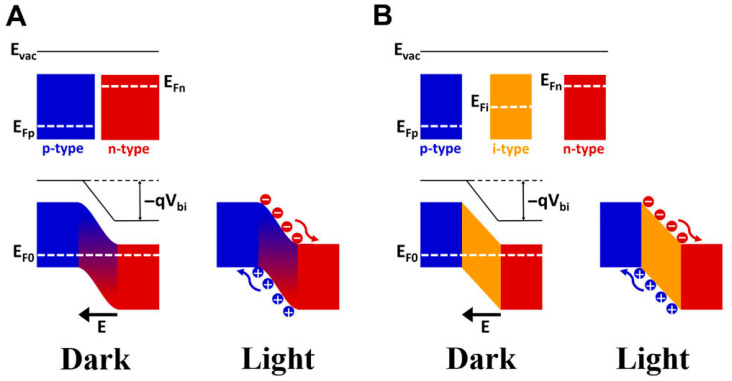
Energy structures of (**A**) p-n junction semiconductors and (**B**) p-i-n junction semiconductors. Reproduced from [86] (*Exploration*, John Wiley & Sons 2022). Licensed under CC BY 4.0.

In general, a photodiode relies on a heterostructure that creates diode characteristics of electrical current, such as p-n/p-i-n structures of heterostructure semiconductors or a metal–semiconductor structure. The junction potential at the interface between p- and n-type semiconductors in a p-n structure (Figure 5A) can be inherently formed by majority carrier diffusion, which is induced by the Fermi level difference. Insertion of an intrinsic semiconductor between p- and n-type semiconductors results in an enhanced depletion region, increasing the photon absorption region, as shown in Figure 5B. The junction potential can also be enhanced by an external voltage bias connected in a reverse way, boosting effective separation of photogenerated electron–hole pairs [72,73,74,75,76]. The reverse bias reduces not only the carrier transit time across the electrodes but also device capacitance due to a widened depletion region, resulting in faster response. Removal of an external voltage from the device circuit leads to the photovoltaic mode, which favors lowering the dark current but suffers from a slow response compared to photodiodes [77,78].

In both photodiode and photovoltaic devices, a region depleted of carriers (depletion region) at the interface can absorb UV light only if its bandgap energy is equal to or greater than the photon energies. Wide-bandgap materials can lend themselves to both the absorption of photon energies greater than 3.1 eV (corresponding to 200 nm) and solar blind response, such as GaN [79,80], AlGaN [81,82], SiC [83], and diamond [84,85].

### 3.3. Photoconductive Detectors for Light Detection

In general, the conductivity of a channel through which current flows can increase when photogenerated carriers begin to be added to it, leading to a current increase in proportion to the light intensity incident on the active area of the device. Photodetectors employing this principle for light detection are called photoconductive detectors or photo-resistive detectors (because resistivity also changes as its reciprocal due to the same reason, i.e., photogenerated carriers) [15,87,88,89]. Photoconductive light detectors take advantage of the photo-induced changes in conductivity in a device. For instance, the abovementioned photodiodes use conductivity increase due to the creation of photogenerated carriers in the depletion region, making them fall within this category (the photoconductive mode) under a reverse bias across heterojunctions such as p-n/p-i-n structures. This external bias voltage would drive photogenerated carriers for photocurrent that increases with light intensity. Another example is a current flow channel of a single semiconductor across which an external bias is applied. When the channel semiconductor absorbs photons, photogenerated carriers increase conductivity, producing a current increase.

**Figure 6:**
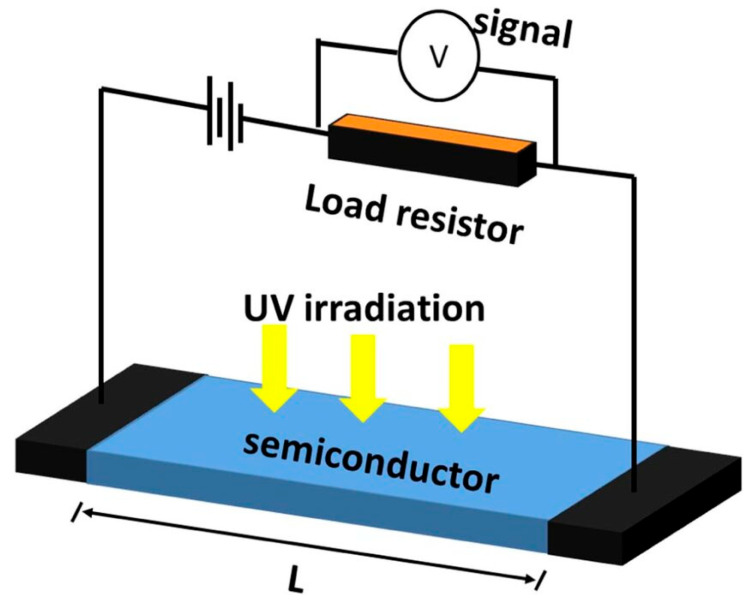
A schematic of a photoconductive UV light detector. The UV light absorption in a semiconductor (Blue) generates charge carriers. Externally connected bias voltage drives movement of the charge carriers so that they are collected at the ohmic electrodes (Black). For a given mobility of semiconductor, both the channel length L and electric field across the electrodes determine the carrier transit time. Reproduced with permission from [98] (*Journal of Physics D: Applied Physics*). Copyright 2014.

Very often, they have non-negligible internal gain for current, for which multiple carriers are collected at electrodes under a device neutrality, given a single pair of photogenerated electrons and holes, particularly when a photogenerated carrier’s lifetime is longer than a carrier transit time [90,91,92]. This eventually increases *R* and *I_d_* while decreasing the device bandwidth. As shown in Figure 6, a semiconductor material is connected between two metal electrodes under an external bias voltage. As photogenerated carriers are generated upon incident light absorption, external bias drives those carriers for current. For UV light detection, wide-bandgap semiconductors can be adopted between device electrodes as a medium where UV photon absorption takes place. This medium also plays a channel through which current flows, meaning its carrier mobility needs to be high for high device bandwidth. The media used for UV photon absorption in photoconductive detectors include ZnO [93], SiC [94], GaN [79], AlGaN [95], gallium oxide (Ga_2_O_3_) [96], Graphene [87], and Si [97].

### 3.4. Field-Effect Transistor-Based Photodetectors

**Figure 7:**
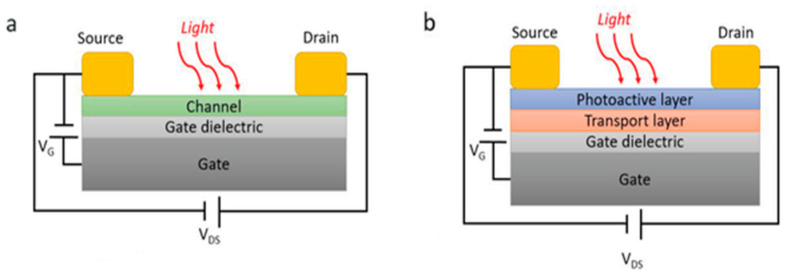
A schematic of two types of FET-based photodetectors. (**a**) The FET where a channel itself absorbs photons. (**b**) The FET where light-absorbing layer is deposited on a channel layer for enhanced light absorption. Reproduced with permission from [105] Advanced Functional Materials, copyright 2020.

Field-effect transistors (FETs) have been used for photodetection via channel conductivity modulation by photogenerated carriers [99]. As shown in Figure 7, its structure typically comprises source, drain, gate, photon-absorbing media (which could also be the same as the channel itself), an oxide dielectric layer (e.g., SiO_2_ or Al_2_O_3_), a channel, and a substrate. Like the role of a gate bias voltage that changes channel conductivity, incident photons can act as a photogate [100]: one type of photogenerated carriers can be trapped in an absorber (in cases of small dimensions such as a quantum dot), near the absorber–channel interface, or channel–dielectric interface. Then, trapped carriers can produce local electric fields as extra gate fields, therefore strongly affecting the gate voltage threshold in FET performance. This eventually enables great change in conductivity to be achieved. Photogenerated carriers themselves in a channel can also contribute to photoconductivity change. FET-based photodetectors take advantage of ultrahigh responsivity, dark current suppression, low power consumption, and compatibility with CMOS processes for integrated chips [101], while facing the challenge of limited bandwidth and degradation issues, particularly in hybrid structures, such as with perovskite and quantum dots [102,103,104].

### 3.5. Avalanche Photodiodes for Light Detection

**Figure 8:**
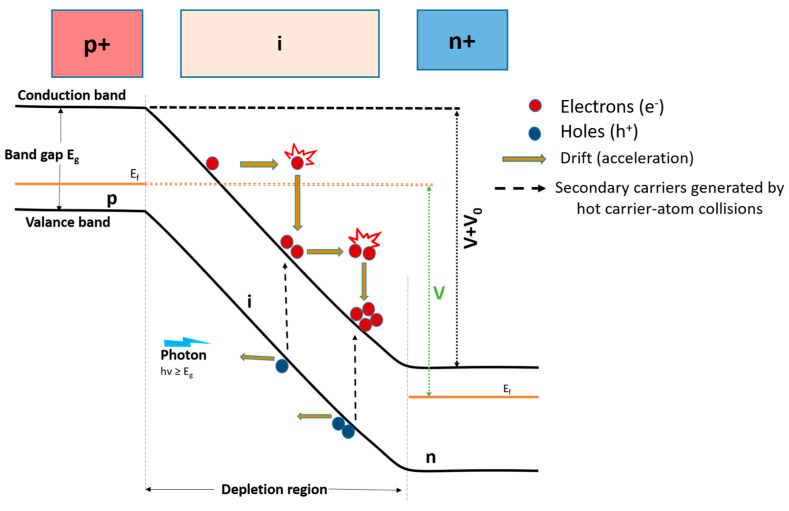
Avalanche effects in a p-i-n structure under reverse bias. V_0_ denotes the inherent junction potential when there is no external bias, while V denotes the external bias voltage.

An avalanche photodiode (APD) utilizes an avalanche effect for which a reverse bias voltage is adjusted to be close to or beyond the breakdown voltage, i.e., tens to hundreds of volts. This high electric field that adds up to the inherent junction field accelerates photogenerated carriers in the junction (carrier depletion) of p-n/p-i-n structure semiconductors, kicking them into (unthermalized) hot carriers with enormous kinetic energies. They collide with lattice atoms, knocking their bound electrons into a conduction band (creating additional free electrons) while generating holes in a valence band. Those additional carriers are again accelerated and undergo similar procedures, giving rise to carrier multiplication in a cascading process, as shown in Figure 8. This avalanche effect leads to an ultrahigh gain of about 10–100 for current, with a consequence of ultrahigh *R*, making the device suited for low-light detection. However, the random characteristics of impact ionization by electron–atom collision produce excess noise as the trade-off for very high gain. Avalanche photodiodes can be operated either in a linear mode or Geiger mode, depending on the external bias magnitude (smaller or greater than a breakdown voltage). SiC is one of the widely used semiconductors as an absorbing medium for UV light APD due to its wide bandgap energy (3.2 eV) and high breakdown voltage (100–400 V for a thin junction, while more than 1 kV for a thick junction) [106,107]. AlGaN and GaN can also be used as a UV light-absorbing medium with additional benefits due to their good solar blindness [108,109]. Si can also be used as an absorbing medium due to its high absorption coefficient at the UV light spectral wavelengths, as mentioned above. However, the very short penetration depth of UV light into Si hinders it from being used as a deep-UV light-absorbing medium due to its surface trap-mediated recombination of photogenerated carriers. Instead, Si-based APD has found application in APD for near-UV light detection [110,111].

## 4. Wide-Bandgap Semiconductors and Metal–Semiconductor–Metal Structure for UV Light Detectors

Photodetectors for UV light detection use wide-bandgap semiconductors as light-absorbing media, such as SiC, ZnO, TiO_2_, GaN, AlGaN, Ga_2_O_3_, AlN, diamond, perovskite oxides (SrTiO_3_, BaTiO_3_), and their hybrid combinations. These materials benefit from the large bandgap energies of 3.2–6.2 eV, solar blindness, and high absorption coefficients at UV wavelengths. Some of them also exhibit the large excitonic binding energies, such as ZnO (~60 meV) [112], AlGaN (~25–80 meV) [113,114], AlN (~71–80 meV) [115,116], and diamond (~70–80 meV) [117,118], enabling the detection of specific UV wavelengths. In addition, among those materials, reasonably high mobility of carriers can also be found in SiC (~800–1000 ${\mathrm{cm}}^{2}$/V·s for electrons, ~100 ${\mathrm{cm}}^{2}$/V·;s for holes) [119,120], GaN (~1000 ${\mathrm{cm}}^{2}$/V·s for electrons, ~200 ${\mathrm{cm}}^{2}$/V·s for holes) [121,122], and diamond (~2200 ${\mathrm{cm}}^{2}$/V·s for electrons, ~1600 ${\mathrm{cm}}^{2}$/V·s for holes) [123,124,125], allowing for high-bandwidth detectors.

**Figure 9:**
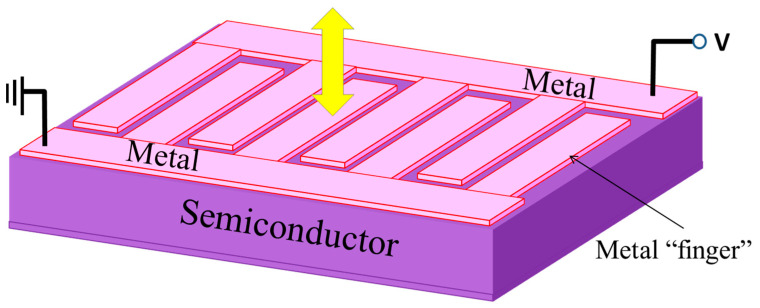
A schematic of an MSM photodetector with interdigitated metal electrodes. The yellow arrow represents the direction of incident light. Reproduced from [131] (Advances in Optical Communications 2014). Licensed under CC BY 4.0.

Wide-bandgap semiconductors are often utilized in “metal-semiconductor-metal (MSM) photodiodes with interdigitated electrodes. They have gained popularity for UV light detection due to their high bandwidth, low dark current, easy fabrication (standard microfabrication techniques), and monolithic integrability with other electronic devices. Interdigitated electrodes of gold or platinum can be patterned like interlaced fingers on a semiconductor (light-absorbing medium), as shown in Figure 9. The metal–semiconductor interface in the MSM structure forms back-to-back Schottky barriers whose height can be modulated by externally applied bias voltage. Interdigitated electrode structures enable the electric fields between electrodes to be homogeneous while the carrier transit time between them would be short, being beneficial for high bandwidth (fast response). An interdigitated electrode structure can also be optimized for dark current suppression and capacitance minimization. As the semiconductors for UV light absorption, GaN [126], AlGaN [127], ZnO [128], SiC [129], and diamond are used [130]. There could be carrier-trapped states at the metal–semiconductor interface affecting I_d_ and the effective bias voltage due to additional local static fields from trapped carriers.

The characteristics of UV light detectors with various wide-bandgap semiconductors are briefly summarized in Table 1. However, it is worth mentioning that wide-bandgap semiconductors still face difficulties in cost-effective production of their crystals [33,132,133], industrial scalability of their crystalline wafer size with high quality [26], and monolithic integration with CMOS-based optoelectronic components [134].

**Table 1:** Wide-bandgap semiconductor-based materials used for UV light detectors.

Active Material	Bandgap Energy (eV)	UV Wavelength (nm)	Photodetector Type	Responsivity (A/W)	Bias Voltage (Volt)	Ref.
AlN	~6.2	193	photoconductive	0.39	20	[135]
Boron Nitride (BN)	~6	200	photoconductive	0.095	20	[136]
Diamond	~5.48	222	photoconductive	22.6	50	[137]
α-Ga_2_O_3_	~5.1–5.3	230	photoconductive	2.71	10	[138]
€-Ga_2_O_3_	~4.9	240	photoconductive	52.77	20	[139]
WO3/AlGaN/GaN	~3.2	240	Photo-FET	1.67 × 10^4^	0.5	[140]
AlGaN	~3.4–6.2	250	photoconductive	~10^6^	5	[141]
Si/SiC	~1.12/3.26	260	Photo-FET	4.63 × 10^5^	5	[142]
TiO_2_	~3.2	260	photoconductive	199	5	[143]
AlGaN/GaN	~3.4–6.2/3.4	265	Photo-FET	3.6 × 10^7^	−8.2	[144]
Graphene/SiC	~0/3.26	275	photoconductive	5.425 × 10^3^	1	[145]
SnO_2_	~3.6	322	photoconductive	1.353 × 10^3^	10	[146]
ZnO	~3.37	325	photoconductive	2.6 × 10^4^	8	[147]
GaN	3.4	340	photoconductive	~1.3 × 10^4^	1	[148]
CsPbCl3 nanowires	~3.03	360	Photovoltaic	0.398	0	[149]
SiC	3.26	360	n^+^/p/n^−^ Photo-FET	2.02 × 10^4^	37	[150]
GaSe/GaN	~3.4	362	p-n heterojunction	1.38 × 10^5^	−4	[151]
W_18_O_49_/TiO_2_	~3.2	365	Photoconductive	1.6 × 10^4^	1	[152]
AlGaN/GaN	~3.4–6.2/3.4	365	Photo-FET	1 × 10^6^	−8.2	[144]

## 5. Silicon-Based UV Detectors: Structural Advancements and Challenges

**Figure 10:**
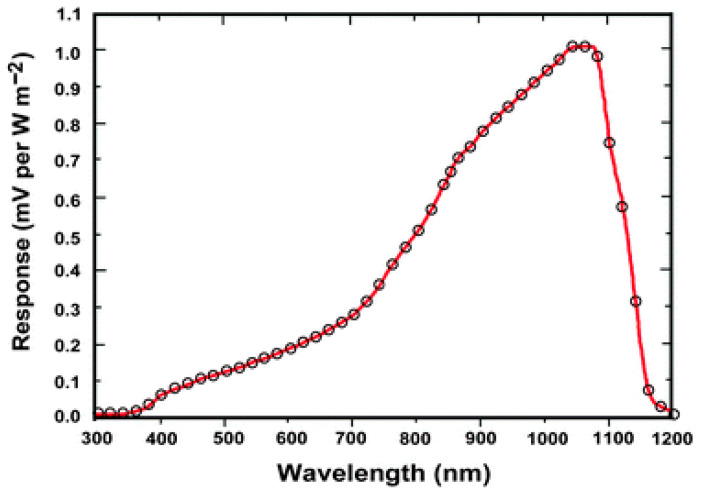
Spectral response curve (Responsivity R versus wavelength) of Si used in solar cells under solar radiation. R drops rapidly at above 1100 nm due to the cutoff given by bandgap energy of 1.12 eV. Reproduced with permission from [153] (Chemical Society Reviews 2013).

The use of silicon for UV photodetectors can offer many advantages. One of them is the availability of mature CMOS-compatible technologies for passivation, metallization, photolithography, and doping. It can also offer cost-effective fabrication of high-quality detectors, large-scale wafer-based production of detector arrays, and integrability with other electronic components. Despite its bandgap energy of 1.12 eV (1100 nm) and indirect bandgap nature, it exhibits large absorption coefficients at UV wavelengths (Figure 2), as mentioned above. However, Si absorbs visible photons in addition to UV photons but shows very low *R*, particularly in the deep UV regions (near 300 nm), as shown in Figure 10. The low sensitivity of Si to UV light is due to shallow penetration depth (a few nanometers) below its surface and significant surface trap-induced non-radiative recombination of photogenerated carriers. Various surface engineering techniques and sensitization strategies could be applied to improve the UV light absorption and reduce the surface-trap-induced non-radiative recombination, finally enhancing the UV detection performance of Si.

### 5.1. Surface Passivation

The shallow penetration depth of UV light into Si leads to the generation of photogenerated carriers, mostly near its surface. The Si surface contains defect states, such as dangling bonds, that are unwantedly created during processes of etching and polishing. These tend to play the surface-trap state as non-radiative recombination centers. The surface-induced effects for non-radiative decay of carriers can be reduced by depositing a thin dielectric layer on the Si surface, such as SiO_2_, Al_2_O_3_, and Si_3_N_4_, subject to its transparency to a given UV wavelength. These passivation effects come into play in various ways. First, such a deposited layer can make a chemical saturation of the surface dangling bonds, which neutralizes the recombination centers (chemical passivation), such as found in the deposition of thermally grown SiO_2_, whose oxygen (O) bonds with unsaturated Si atoms [154,155]. Second, the dielectric layers can possess fixed charges, such as negative fixed charges in an atomically deposited layer of Al_2_O_3_ on Si [156,157]. These fixed charges generate surface-normal electric fields and then create a space charge layer near the interface, which is depleted of carriers. This eventually reduces electron–hole recombination rates, called electric field passivation. The abovementioned Si passivation technologies with SiO_2_ and Al_2_O_3_ are widely reported for Si-based solar cells. Si passivation for UV light detection has yet to be used to develop a method that achieves a sufficiently high EQE and requires an understanding of its working principle, including the relevant carrier dynamics.

A surface passivation approach suggested by Nizdak et al. [158] involved delta-doped surface passivation using molecular beam epitaxy (MBE). For such passivation, a single atomic layer of boron was deposited on a Si substrate, followed by epitaxially overgrown Si that creates a stable dipole and effectively passivates the interface traps. This process enabled suppression of surface recombination and eliminated the charge-induced instabilities of Si. Further deposition of multiple delta-doped layers was performed to control the band bending and directly extend the depletion region to the Si surface for efficient carrier collection. This led the device to achieve a stable high EQE of 80% at a 206 nm UV wavelength, which corresponds to a responsivity of 0.1329 (A/W).

### 5.2. Ion Implantation

**Figure 11:**
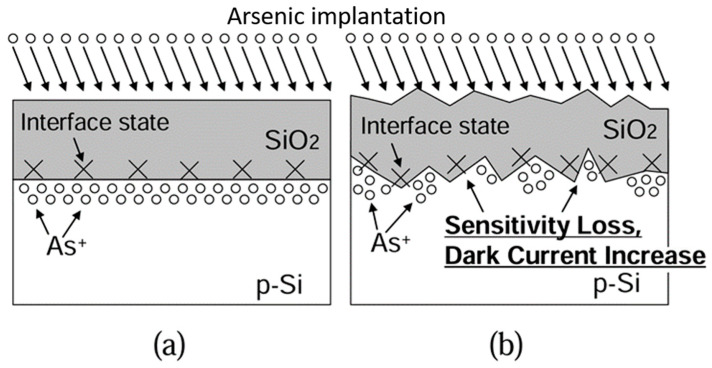
A schematic of cross cross-section of thin n^+^ layer formed on (**a**) atomically flat Si surface and (**b**) conventional Si surface. Reproduced from [160] (J-STAGE, ITE Transactions on Media Technology and Applications 2014) (free access).

Unwanted carrier recombination near the surface can also be reduced by implanting external ions or distributing impurities near the surface region of pure Si wafers in the form of vacancies and interstitials, benefitting the successful collection of UV-photogenerated carriers [159]. For instance Kuroda et al. [160] achieved approximately 100% internal QE with the estimated *R* of 0.2016 (A/W) under 250 nm UV illumination using a Si photodiode fabricated with low-energy ion implantation in the following way: the p-type Si wafer was initially annealed at high temperature both to flatten its surface at an atomic level and to reduce the surface trap states at an interface between Si and SiO_2_ layer; the oxidized layer already existed or generated during annealing process. Figure 11a presents a cross-sectional view of a p-type Si wafer with an atomically flat surface achieved by standard RCA cleaning followed by annealing of the Si wafer. Figure 11b shows a conventional Si surface with a rough surface (cross-sectional view). Subsequently, low-energy arsenic ion implantation introduced donor impurities, creating an n-type region near the surface so that a charged spacer layer forms due to positively ionized donors (As+). Therefore, the induced electric fields hindered the carrier recombination and improved the internal QE of the photodetector. The device showed stable performance under prolonged (1000 min) UV illumination.

**Figure 12:**
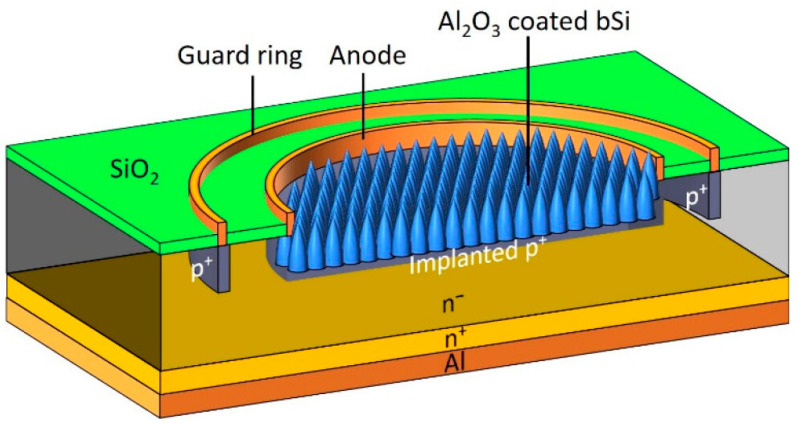
Boron-implanted b-Si photodiode. The SiO_2_ layer serves as masking material. Reproduced from [161] (ACS Photonics 2023) Licensed under CC-BY 4.0.

Low-energy boron implantation was conducted on the plasma-etched surface of black silicon (b-Si) [161] to generate the 1.5 μm deep p-type region near the surface, as shown in Figure 12. Near the surface, the ionized acceptors (implemented boron ions) built up surface-normal electrical fields together with ionized donors of the n-type Si, generating a surface-near region depleted of carriers. This eventually reduces the carrier recombination rate. Carrier recombination rate near the surface is further reduced by atomically layered deposition of a 50 nm Al_2_O_3_ film, as mentioned above. This structure yields *R* of 0.15 (A/W) at 200 nm wavelength and 0.3 (A/W) at 400 nm. A similar device designed by Garin et al. [162] achieved EQE of 132% with a corresponding *R* value of 0.213 (A/W).

### 5.3. Quantum Cutting

The principle of quantum cutting is straightforward yet profound. A fluorescent material, immobilized on the surface of Si in a photodetector, absorbs high-energy UV photons and emits two or more photons with lower energy through a cascade of energy transitions of downconversion [163]. In such a scenario, the emitted photon energy should fall within the detection range of the Si photodetector. The downconversion by photon splitting, called “quantum cutting”, is theoretically expected to achieve a high EQE exceeding 100% since a single UV photon can lead to the generation of multiple electron-hole pairs in the underlying Si substrate.

The implication of quantum cutting in UV detecting devices relies on the development of efficient down-conversion materials. Lanthanide-doped phosphors [163,164], Silicates [165], and perovskite nano-crystals and quantum dots [166,167] have recently garnered attention as downconversion materials. Lanthanides have the unique energy levels of a ladder pattern for 4f electrons, which are shielded by 5s/5p orbitals, making them less vulnerable to host lattice vibration. This leads to long lifetimes of excited states and enhanced radiative decay. Lanthanides can serve both as sensitizers that absorb UV photons and as activators that are excited and emit photons of lower energies (near-infrared wavelengths). Quantum cutting materials are directly applicable using conventional coating methods or can be designed with luminescent concentrators (i.e., waveguide) to collect emission light effectively upon UV absorption in the 200 nm to 400 nm range [153,168].

For Si-based detectors, Shao et al. spin-coated the compound materials, i.e., La^3+^, Yb^3+^ co-doped CsPbClBr_2_ quantum dots, on Si and achieved an EQE of 55.6% and an *R* of 0.131 (A/W) at the UV wavelength of 240 nm [169]. The La^3+^ ions reportedly suppress the non-radiative decay by reducing the defect, whereas the Yb^3+^ helps the UV photon down-conversion to near-infrared photons, as in the case of CsPbCl_3_. An *R* of 0.14 A/W at approximately 300 nm UV is achievable [170]. An R of 0.006 A/W by Dy^3+^-doped CsPbCl_2_Br_1_ [171] and an R of 0.009 (A/W) for Eu^3+^-doped CsPbCl_2_Br_1_ [172] quantum dots on Si under UV 320 nm radiation have also been reported.

Despite the encouraging results mentioned above, the practical implementation of quantum cutting in Si-based photodetectors remains challenging. The optimization of dopant concentration and achieving high efficiency in energy transfer between sensitizers and activators is not straightforward. For quantum dots, their long-term stability and the environmental factors also remain key concerns. The development of more robust quantum cutting materials in the future could pave the way for next-generation and highly sensitive Si-based photodetectors.

### 5.4. Surface Etching and Plasmonic Surface Nano-Structuring

The UV light sensitivity of Si can be enhanced through surface etching, which produces micro- and nano-scaled texturing, such as pores, cones, and pyramids. Microscale roughness of the Si surface can reduce the surface reflections, thus making absorption more likely to take place. Nanoscale roughness may create quantum confinement effects in Si, with a consequence of widening effective bandgap energies and corresponding enhancement of UV photon absorption [173]. Furthermore, reflected photons from one micro- and nano-scaled structure in a random direction can be absorbed by neighboring micro- and nano-scaled structures, resulting in strong light absorption [174]. However, photogenerated carriers due to such photons absorbed in shallow regions of locally textured structures suffer carrier recombination due to defects before carrier collection at electrodes [175].

A UV photodetector based on surface-etched n-type Si has been reported by Asad et al. [176]. They performed electrochemical etching of the Si surface, where electrical current modulated the etching ratio, under UV light illumination (here, UV light for etching purposes). Then, the etching-generated nano-porous Si was treated with Nd:YAG nanosecond pulsed laser for structural improvement and recrystallization. The photodetector utilized interdigitated Pt electrodes in the MSM structure mentioned above, and the *R* for detecting 380 nm UV light was estimated as 2.01 (A/W).

Gold (Au) nanoparticles deposited over the porous surface could further increase *R* due to surface plasmon resonance (SPR) effects. The collective oscillation of conduction electrons on Au nanoparticles can occur coherently with incident light of a specific wavelength [177,178,179]. SPR leads to strong absorption and elastic scattering of light, generating enhanced local fields around the particles [180,181]. By carefully controlling the size and shape of plasmonic nanoparticles, the UV absorption can be enhanced [182]. Ismail et al. [183] recently reported the surface etching followed by the thermal oxidation of Si. Au nanoparticles were immobilized on the etched surface, enabling *R* of 0.205 (A/W) to be achieved for detecting UV light at 365 nm.

**Figure 13:**
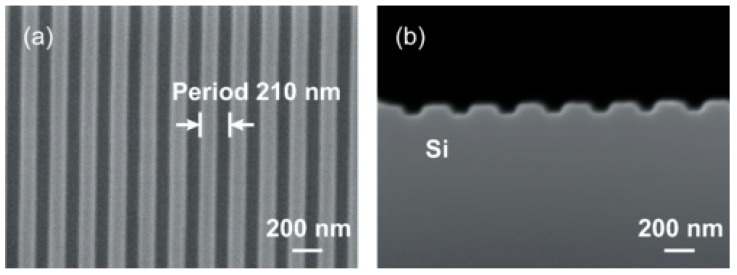
SEM images of periodic corrugation on Si surface. (**a**) Top view and (**b**) cross-sectional view. Reproduced from [184] (Physical Review Letters 2025); licensed under CC BY 4.0.

Interestingly, silicon alone can simultaneously act as both a plasmonic resonator and an active absorbing medium, as demonstrated in a recent experiment by Tanaka et al. [184]. They used electron-beam lithography followed by reactive ion etching to carve periodic corrugations with a pitch of 210 nm on an n-type Si substrate, as shown in Figure 13. The corrugated structure of Si enabled the SPR effects to be activated without using noble metals at deep UV wavelengths since Si had the negative values of the relative permittivity at those wavelengths, such as 266 nm. SPR effects produced enhanced local fields near the surface, leading to improved absorption and, consequently, enhanced efficiency in the photogeneration of carriers. The device showed *R* of 0.18 A/W and 0.3 A/W at 266 nm at bias voltages of 7 V and 10 V, respectively. This demonstration of SPR-induced amplification of the E field with a nanoscale grating offers new routes for CMOS-compatible Si UV photodetectors.

### 5.5. Wide-Bandgap Semiconductor Integrated Si

Wide-bandgap semiconductors that are suited not only for UV light absorption but also for solar-blind applications can be deposited over the Si surface to construct hybrid heterostructures. Here, wide-bandgap semiconductors serve as a medium where UV photon absorption and subsequent carrier generation take place, whereas the Si, the underlying layer, acts as a carrier capture and transport layer. This hybrid structure can provide the advantages of better selectivity for UV light absorption and effective passivation of Si, which may reduce the carrier recombination rate. Basically, this hybrid structure still possesses advantages of using wide-bandgap materials such as the suppression of dark current due to fewer number of carriers excited at room temperature, availability of high applied bias due to high breakdown voltage, resistance against radiation damage, and chemical/thermal/mechanical robustness in addition to the aforementioned solar blindness and high efficiency of UV photon absorption [134,185,186,187,188,189].

However, it is worthwhile to note that this hybrid structure faces several challenges, including lattice mismatch-induced dislocation of crystals [190,191,192], thermal expansion mismatch [193,194,195], hybrid interface-induced defects [196,197], and the requirement of a (wide-bandgap) crystal growing temperature too high to be compatible with Si-based CMOS processes [198,199]. The crystal dislocations in such a hybrid structure lead to high etch pit density, causing non-radiative recombination centers to be generated for high dark current and degrading photodetectors over time. Thermal expansion mismatch can also develop unwanted strains during crystal growing at high temperatures or annealing, disabling efficient wafer-scale fabrication [200]. To circumvent the aforementioned challenges related to lattice and thermal mismatch, a suite of sophisticated defect and strain engineering strategies is being applied by researchers. One method is strain management through buffer layer engineering on the Si surface, which serves as a foundation for wide-bandgap materials [201,202,203,204]. The buffer layer, which is usually graded, absorbs the stress and manages the mismatch during the growth of wide-bandgap materials by bending and trapping the dislocations. Consequently, the dislocation density in the active area decreases, which leads to improved performance of the device. Another method is interfacial chemical engineering, which involves the creation of a spotless atomic interface by removing the native contamination from the Si surface, such as SiO_2_, through chemical reactions and replacing it with the desired oxides of the same family of the active material considered for deposition [205,206]. This oxide layer with intermediate lattice parameters acts as a template for the subsequent deposition of wide-bandgap materials.

In addition, the inherent surface properties found in wide-bandgap semiconductors alone, such as surface defects and Fermi energy pinning, may need to be considered when they are brought into contact with Si to avoid substantial surface leakage current due to additional passivation [207,208,209,210,211,212,213,214]. Table 2 displays the performance of UV photodetectors based on Si, including wide-bandgap semiconductor-integrated Si, operated at various UV wavelengths. In some cases, the EQE, which exceeds 100%, indicates photoconductive gain more than unity, such as in avalanche multiplication.

**Table 2:** Performance of Si-based UV light detectors at various UV wavelengths.

Material	Device Type	Wavelength nm	EQE %	Responsivity (A/W)	Refs.
Boron implanted n-type black Si	p-i-n diode	200	100	0.15	[161]
n-type black Si	p-i-n diode	200	132	0.213	[162]
p-type Si	p-n diode	206	80	0.13	[158]
β-Ga_2_O_3_/p-type Silicon	MSM Schottky diode	230	378.5	0.702	[206]
La^3+^, Yb^3+^ co-doped CsPbClBr_2_/Si	p-n diode	240	55.6	0.13	[169]
p-type Si	n^+^–p–n diode	250	100	0.2016	[160]
SiC/Si	MSM Schottky diode	250	89.3	0.18	[215]
β-Ga_2_O_3_/p-type Si	MSM Schottky diode	250	19,250	38.8	[216]
β-Ga_2_O_3_/AiN/p-type Si	MSM Schottky diode	250	5880	11.84	[216]
SiC/p-type Si	MSM Schottky diode/MOSFET	260	2.2 × 10^8^	4.63 × 10^5^	[129]
Corrugated n-type Si	photoconductor	266	140	0.3	[184]
AlGaN/Si	p-i-n diode	274	54.3	0.12	[201]
La^3+^, Yb^3+^ co-doped CsPbCl_3_/Si	photodiode	300	57.9	0.14	[170]
Eu^3+^-doped CsPbCl_2_Br_1_/Si	photo diode	320	3.41	0.009	[172]
Dy^3+^-doped CsPbCl_2_Br_1_/Si	photo diode	320	2.3	0.006	[171]
GaN/Si	Asymmetric MSM diode	340	21,200	58	[217]
ZnO/Si	heterojunction photoconductor	350	24,100	68	[218]
Porous n-type Si	MSM diode	380	658	2.01	[176]
Au NPs/porous p-type Si	MSM diode	365	69.69	0.205	[183]
Boron-implanted n-type black Si	p-i-n diode	400	100	0.3	[161]

### 5.6. Comparative Analysis

The establishment of Si-based stable and standard photodetectors requires careful consideration of the balance between their performance and industrial viability. Among the abovementioned techniques, the surface passivation and external ion implantation techniques are industrially scalable with long-term stability and are compatible with CMOS technology despite the modest gains in responsivity in the UV region. Considering an enhanced performance, wide-bandgap materials integrated with Si offer high responsivities with inherently robust stability. Although their deposition methods are also well-established, their cross-contaminations pose a threat to full-scale CMOS compatibility, as the CMOS foundries require extreme purity. The thermal and lattice mismatches between the wide-bandgap materials and Si could also lead to performance degradation over time, reducing the overall effectiveness of the photodetectors. UV photodetectors based on the quantum cutting mechanism, despite being a novel method of photon down-conversion to measure UV light intensity, also face severe limitations in industrial-scale production and CMOS incompatibility issues due to contamination. The poor stability of the fluorescent materials is still evolving.

Surface etching of Si also boosts its sensitivity in the UV region. However, it calls into question the large-scale uniformity. Large-scale etching has its own scaling issues that introduce non-uniformity in etching across the area, which leads to non-reproducible performances of the final devices. The plasmonic nano-structuring to create a corrugated Si surface through electron beam lithography could be proven useful in full-scale integration into CMOS technology, but with a large amount of energy required for the lithography device. Further research is needed to improve the responsivity to even higher levels.

## 6. Conclusions

Ultraviolet photodetectors based on wide-bandgap semiconductors have typically been reported due to their many advantages, such as high responsivity, solar blindness, chemical/thermal/mechanical robustness, and good tolerance against radiation damage. However, they are expensive to produce in high crystal quality and incompatible with Si-based CMOS processes, all while quite often having inherently rich surface defect states. Alternatively, the use of Si as an active medium ensures Si-based CMOS compatibility, allowing for the easy integration of Si-based UV detectors with other electronic devices into a chip. The significant absorption coefficients of Si at UV wavelengths can also support the use of Si as an active medium. However, the penetration depth of UV light into the Si surface is notably limited, rendering surface-near photogenerated carriers to encounter the surface trap states. This eventually hinders the effective transport of photogenerated carriers to electrodes. Recently reported advancements in surface passivation, ion implantation, quantum cutting, surface nano-texturing, and hybrid integration with wide-bandgap materials have improved Si photodetectors’ responsivities up to a considerable level. In particular, Si integration with wide-bandgap semiconductors can be regarded as viable for near-future commercialization given the fact that the aforementioned surface treatment/engineering can also be applied to the hybrid structure of Si-wide-bandgap semiconductor to maximize the expected benefits mentioned above. 

## Data Availability

No new data were created or analyzed in this study.
